# Women's decision-making regarding risk-stratified breast cancer screening and prevention from the perspective of international healthcare professionals

**DOI:** 10.1371/journal.pone.0197772

**Published:** 2018-06-01

**Authors:** Linda Rainey, Daniëlle van der Waal, Louise S. Donnelly, D. Gareth Evans, Yvonne Wengström, Mireille Broeders

**Affiliations:** 1 Radboud Institute for Health Sciences, Radboud University Medical Center, Nijmegen, The Netherlands; 2 Prevent Breast Cancer Research Unit, The Nightingale Centre, Manchester University NHS Foundation Trust, Wythenshawe, Manchester, United Kingdom; 3 Genomic Medicine, Division of Evolution and Genomic Sciences, Manchester Academic Health Sciences Centre, Manchester University NHS Foundation Trust, Manchester, United Kingdom; 4 The Christie NHS Foundation Trust, Withington, Manchester, United Kingdom; 5 Department of Neurobiology, Care Sciences and Society, Division of Nursing, Karolinska Institutet & Theme Cancer, Karolinska University Hospital, Huddinge, Sweden; 6 Dutch Expert Centre for Screening, Nijmegen, The Netherlands; UNM Cancer Center, UNITED STATES

## Abstract

**Introduction:**

Increased knowledge of breast cancer risk factors may enable a paradigm shift from one-size-fits-all breast cancer screening to screening and subsequent prevention guided by a woman’s individual risk of breast cancer. Professionals will play a key role in informing women about this new personalised screening and prevention programme. Therefore, it is essential to explore professionals’ views of the acceptability of this new programme, since this may affect shared decision-making.

**Methods:**

Professionals from three European countries (the Netherlands, United Kingdom, and Sweden) participated in digital concept mapping, a systematic mixed methods approach used to explore complex multidimensional constructs.

**Results:**

Across the three countries, professionals prioritised the following five themes which may impact decision-making from the perspective of eligible women: (1) Anxiety/worry; (2) Proactive approach; (3) Reassurance; (4) Lack of knowledge; and (5) Organisation of risk assessment and feedback. Furthermore, Dutch and British professionals expressed concerns regarding the acceptability of a heterogeneous screening policy, suggesting women will question their risk feedback and assigned pathway of care. Swedish professionals emphasised the potential impact of the programme on family relations.

**Conclusions:**

The perspectives of Dutch, British, and Swedish professionals of women’s decision-making regarding personalised breast cancer screening and prevention generally appear in line with women’s own views of acceptability as previously reported. This will facilitate shared decision-making. However, concerns regarding potential consequences of this new programme for screening outcomes and organisation need to be addressed, since this may affect how professionals communicate the programme to eligible women.

## Introduction

The current one-size-fits-all breast cancer screening strategy is effective in reducing breast cancer mortality, however increased knowledge of breast cancer risk factors may enable a more personalised approach [1,2]. Defining screening regimens based on breast cancer risk is expected to optimise the balance between the known benefits and harms of screening [3]. Risk-stratified screening delivery enables the determination of a more favourable screening interval, age range, and screening modality for women at varying levels of breast cancer risk. Furthermore, the existing screening infrastructure provides a valuable opportunity to educate women on ways to actively reduce their risk, ultimately aiming to prevent breast cancer [4]. These promising prospects have led to the initiation of several large prospective studies developing breast cancer risk prediction models to guide screening and prevention on a population level. However, successful implementation of risk-based breast cancer screening and prevention is contingent on the acceptability of the programme, both from the perspective of eligible women and professionals.

Although integrated risk-based screening and prevention is not yet practised, several studies have piloted breast cancer risk and prevention counselling in primary care settings. This has led to the identification of considerable practical implications, e.g. time constraints and a great need for additional [5–8]. Primary care professionals consistently reported a lack of knowledge of all aspects of personalised screening and prevention [9–11]. Previous experience with the prescription of preventative measures and having women show interest by initiating the conversation about breast cancer risk facilitated acceptance, emphasising a shared decision-making process [9–11].

Healthcare professionals will play a key role in informing women about personalised breast cancer screening and prevention. Although it has not yet been decided which healthcare professionals will be involved in risk-based screening and prevention, it is conceivable that, for example, radiologists, radiographers, oncologists, and GPs will encounter women with questions about participation. Their communication about the programme will be affected by whether they perceive it to be acceptable, both in light of their personal day-to-day work activities and considering eligible women who may participate in the programme. It is important that professionals prioritise women’s needs, since it is women who are likely to benefit from a personalised screening and prevention approach. Women have previously spoken about requiring additional knowledge about breast cancer risk to be able to decide on participating in the programme [12]. They have also emphasised that they need a knowledgeable professional to inform them about their risk [13]. In general, women welcome breast cancer risk communication as they feel it allows for a more proactive approach to breast cancer prevention [14,15]. However, they have expressed concerns about subsequent anxiety and the potential for stigma [16,17].

To explore whether professionals’ views are in line with those of women, we have asked professionals to consider risk-based breast cancer screening and prevention from the perspective of eligible women to evaluate acceptability. This will provide insights into professionals’ underlying beliefs and concerns regarding the programme. Professionals from three of the countries that are currently developing a breast cancer risk prediction model participated, i.e. the Netherlands, the United Kingdom, and Sweden. The resulting overview of perceptions will be a valuable support tool to address the topic of personalised breast cancer screening and prevention with professionals, health policy makers, and eligible women, taking into account potential cultural variation across the three countries.

## Methods

### Study design

Professionals participated in digital concept mapping. We opted for concept mapping, because it offers a systematic, time efficient way for our international healthcare professionals to explore complex multidimensional constructs using a mixed-methods approach [18]. Ethics approval was acquired from the regional ethics committee CMO Arnhem-Nijmegen in the Netherlands, Health Research Authority in the United Kingdom, and the Regional Ethical Review Board at the Karolinska Institutet Stockholm in Sweden. All participants provided informed consent before the start of the study.

### Participants

In total, 162 professionals were invited to participate in the study: 28 professionals in the Netherlands, 59 in the UK, and 75 in Sweden. Convenience sampling was used to invite participants who were part of the professional network of some of the researchers (MB, GE, YW, AJ). Invitations were sent until at least ten professionals per country participated. Participants were invited based on their extensive knowledge of breast cancer screening and/or prevention, and their varied professional backgrounds and practice settings (i.e. screening, primary care, hospital). Participants were not necessarily aware of risk-based screening and prevention prior to participation in the study. This enabled them to explore the concept without preconceived ideas, which allowed for more diversity in participants’ perspectives. All participants were practising their profession at the time of the study.

### Procedure

Concept mapping includes seven structured steps: (1) define participants; (2) formulate the seeding statement; (3) brainstorm: generate statements; (4) sort statements; (5) rate statements; (6) analyse statements; and (7) interpret concept maps [18]. Steps 3 to 6 were performed digitally using concept mapping software (Concept Systems Incorporated). The participants performed steps 3 to 5, whereas steps 1, 2, 6, and 7 were performed by the researchers. Participants were informed of each step by email, which also contained a link to the concept mapping website and personal log-in details.

To obtain a broad range of perspectives, we asked each participant in all three countries to individually brainstorm for ten minutes using a general seeding statement: ‘thinking as broadly as you can, generate statements on perceptions (e.g. barriers/worries/fears/advantages/facilitators) that women can have of personalised risk-based breast cancer screening and primary prevention’. Participants in the Netherlands were provided with the seeding statement in Dutch, all other participants were asked to perform the task in English. The statements of all participants were collected and sorted per country by the researchers. Duplicate statements were removed.

Next, we presented each participant with all unique verbatim statements generated by all other participants of their respective country, e.g. each Swedish participant was presented with the statements generated by all other Swedish participants. Participants were then asked to sort these statements according to theme, based on their personal judgement, and label each category. There was no limit to the number of categories that could be generated.

Finally, each participant was asked to rate the statements generated by all participants of their respective country on a scale from 1 to 10, using the following question: ‘take the perspective of a woman eligible for the national breast cancer screening programme, how important is this statement for a woman’s consideration to participate or not participate in a personalised risk-based breast cancer screening and prevention programme?’.

Additionally, participants provided information on some personal characteristics (i.e. age, gender, profession, and years of work experience). Participant data was included for analysis if at least 105 statements were categorised and at least one statement was rated, based on the requirement settings of the concept mapping software.

### Data analysis and interpretation

The concept mapping software uses multidimensional scaling and cluster analysis to identify patterns in participants’ sorted and rated data, which are graphically represented by two-dimensional cluster rating concept maps. The overall fit of the concept map is described by a stress-value, which compares the obtained concept map to the dissimilarity matrix that served as input [19]. A stress-value between 0.21 and 0.37 represents sufficient fit, where a lower value within said interval is considered most optimal [19].

A cluster rating map was created for each country displaying clusters with statements similar in thematic content. Additionally, the software calculated the average priority rating of statements within each cluster, which was graphically depicted by assigning layers to each cluster, with more layers representing higher priority of the statements within that cluster. The software labelled each cluster based on the labels previously assigned by participants, through word pattern analysis. To obtain the concept map which best fit the data, both the minimum (5) and maximum (20) number of clusters able to describe the data were explored, i.e. the most general and specific content analysis, respectively. These cut-off values are determined by the concept mapping software, which does not facilitate fewer than 5 or more than 20 clusters. Therefore, each cluster rating map started with 20 clusters, after which a researcher (LR) evaluated the content of each cluster and calculated the percentage of items within each cluster that corresponded to the cluster label assigned by the software programme. Next, the number of clusters was reduced one at a time until the data was represented by 5 clusters. At each step the percentage of statements that corresponded to the category label assigned by the software was calculated (by LR). If the thematic content of statements within one cluster was relatively homogeneous, but not adequately represented by the label, a new label was manually assigned. The final cluster count per country was based on the number of clusters that resulted in the highest percentage of statements corresponding to each cluster label. Although the main cluster analysis was performed by one researcher (LR), other researchers (DvdW, MB) were consulted in case of ambiguity.

Additionally, we explored whether type of profession influences participants’ perspectives, distinguishing between clinicians and ‘other professionals’ (i.e. researchers and ‘other’) using pattern match and Pearson correlation coefficients. Correlation coefficients were classified according to the following guidelines: <0.5 low, 0.5–0.7 moderate, 0.7–0.9 high, and 0.9–1.0 very high positive correlation [20].

The cluster rating maps representing the perspectives of participants from the Netherlands, UK, and Sweden were compared to explore similarities and differences. Perspectives of the Dutch, British, and Swedish participants were integrated by selecting the five themes with the highest average priority rating across the three countries.

## Results

### Participant characteristics

A total of 48 participants were assigned to the brainstorm task: 19 Dutch professionals (response rate: 68%), 16 British professionals (response rate 27%), and 13 Swedish professionals (response rate 17%). Ultimately, 44 participants completed all three tasks, with 4 (8.3%) professionals dropping out at different stages due to the required time investment of around 90 minutes. Table 1 provides an overview of participant characteristics and sample sizes per concept mapping task and participating country. Most of the participants in all three countries were middle-aged females with a varied professional background and over 10 years of experience in the field.

**Table 1:** Characteristics of the participating professionals per country.

Task participants	Netherlands		United Kingdom		Sweden	
Total invited (n)	28		59		75	
Participated brainstorm (n)	19		16		13	
Unique statements generated (n)	124		109		75	
Participated sort^a^ (n)	17		14		11	
Participated rate^b^ (n)	17		15		12	
**Personal characteristics**						
Gender (% female)	84.2		75.0		66.7	
Age (median in years, range)	54	[35–68]	48	[24–74]	45	[29–64]
Profession (n, %)						
Researcher	7	(40)	3	(18)	5	(45)
Clinician	5	(27)	9	(53)	6	(54)
Other^c^	5	(33)	5	(29)	1	(1)
Years of experience (median, range)	21	[8–37]	13	[2–48]	11	[5–38]

^a^ participants that met the threshold criterion of having sorted at least 105 statements

^b^ participants that met the threshold criterion of having rated at least one statement

^c^ other professions included, e.g. dietician, manager screening unit, coordinator screening unit

### Brainstorm: Generate statements

In response to the seeding statement, Dutch professionals generated a total of 124 unique statements, British professionals 109, and Swedish professionals generated 75 unique statements. S1 Table provides an overview of all generated clusters and statements, including the average priority ratings, stratified by country.

### Sort and rate statements

#### The Netherlands

**Fig 1:**
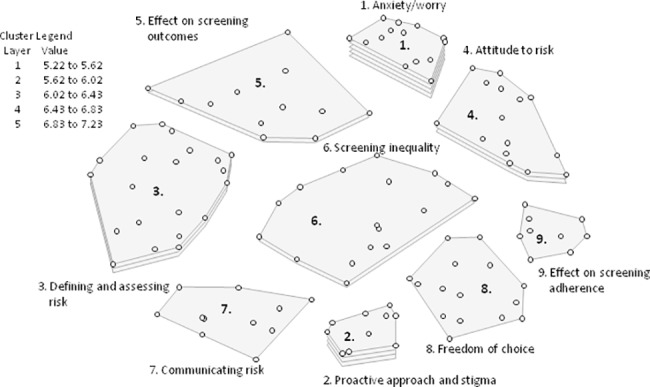
Cluster rating map the Netherlands^a^. ^a^Each cluster rating map displays clusters containing statements (the dots) that are similar in thematic content. The layer(s) of each cluster represents the average priority ratings of the statements within that cluster, more layers signify higher priority. The legend provides an indication of the average priority ratings that the statements within each cluster received.

The number of categories generated by Dutch participants ranged from 4 to 24 (median = 9). Average statement ratings varied from 3.17 to 8.28, representing the statement which was rated least important, i.e. ‘who has the time to participate in more frequent screening?’, and most important, i.e. ‘a woman at high risk of developing breast cancer wants a shorter screening interval’, respectively. The concept map of the Dutch professionals generated a stress-value of 0.34, indicating sufficient fit. Fig 1 presents the cluster rating map of Dutch professionals.

The cluster rating map providing the best fit contained the following nine categories: (1) Anxiety/worry; (2) Proactive approach and stigma; (3) Defining and assessing risk; (4) Attitude to risk; (5) Effect on screening outcomes; (6) Screening inequality; (7) Communicating risk; (8) Freedom of choice; and (9) Effect on screening adherence. The category ‘Anxiety/worry’ was considered most important with an average rating of 7.23, and includes statements such as: ‘Increasing the screening frequency provides insecurity’, ‘Knowing you’re high risk instils anxiety’, and ‘Having an increased risk due to non-modifiable risk factors will increase anxiety and worry’. ‘Communicating risk’ was rated least relevant to women’s decision-making process with an average rating of 5.22. This category contains statements such as: ‘Information about preventative measures needs to be relayed by general practitioners’, ‘Information about preventative measures needs to be relayed by clinicians’, and ‘There is no clear message about preventative behaviours like diet and exercise’.

A comparison of the rating patterns of Dutch clinicians (n = 5) versus other Dutch professionals (n = 12) showed strong correlation (*r* = 0.78). Both groups prioritised ‘Proactive approach’ and ‘Stigma’ in women’s decision-making process. However, clinicians valued the importance of ‘Communication’ more than other professionals, rating it as the sixth most important theme (out of nine), whereas other professionals assigned ‘Communication’ the lowest rating.

#### United Kingdom

**Fig 2:**
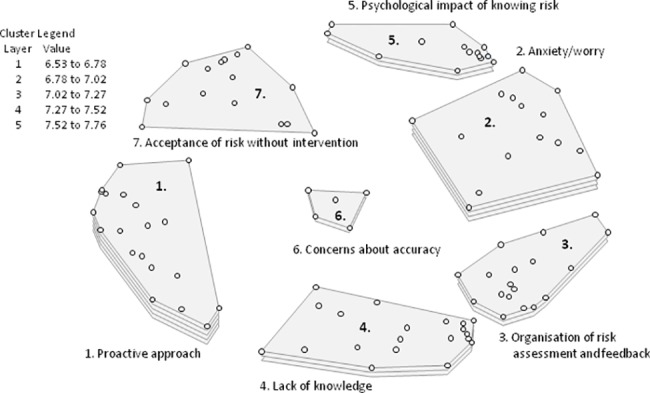
Cluster rating map United Kingdom^a^. ^a^Each cluster rating map displays clusters containing statements (the dots) that are similar in thematic content. The layer(s) of each cluster represents the average priority ratings of the statements within that cluster, more layers signify higher priority. The legend provides an indication of the average priority ratings that the statements within each cluster received.

British participants generated between 5 and 26 categories (median = 8). Average statement ratings varied from 4.00 (statement: ‘Is personalised risk-based breast cancer screening and primary prevention performed in other countries’) to 9.13 (statement: ‘This may help my children or other relatives; if my risk is high, theirs could be too’). The overall fit of the British concept map was sufficient with a stress-value of 0.29. Fig 2 presents the cluster rating map of British professionals.

The cluster rating map which best fit the data contained the following seven categories: (1) Proactive approach; (2) Anxiety/worry; (3) Organisation of risk assessment and feedback; (4) Lack of knowledge; (5) Psychological impact of knowing risk; (6) Concerns about accuracy; and (7) Acceptance of risk without intervention. British professionals regarded the category ‘Proactive approach’ as most important to a woman’s decision-making process of participating in personalised screening and prevention (average rating = 7.76). This cluster includes statements such as: ‘I can take control of some of my own risk factors and take steps to reduce them’ and ‘I want to do everything I can to avoid breast cancer’. ‘Acceptance of risk without intervention’ was considered least relevant (average rating = 6.53). Statements clustered within this theme, include: ‘I can't change it anyway, so why find out what the risk is?’, ‘I feel it’s fate and I don't want to know my risk’, ‘I don't feel any extra benefit from knowing my risk; ‘I won't change my behaviour so what is the point’, and ‘I just want to live a normal life’.

A comparison of the rating patterns of British clinicians (n = 9) versus other British professionals (n = 6) showed a low correlation (*r* = 0.46). Although both groups prioritised ‘Proactive approach’ and ‘Anxiety/worry’, there were large discrepancies in the prioritisation of the other themes. Clinicians prioritised ‘Lack of knowledge’ and ‘Concerns about accuracy’, whereas other professionals prioritised ‘Organisation of risk assessment and feedback’ and ‘Psychological impact of knowing risk’. Conversely, ‘Concerns about accuracy’ was considered the least important theme by the other professionals.

#### Sweden

**Fig 3:**
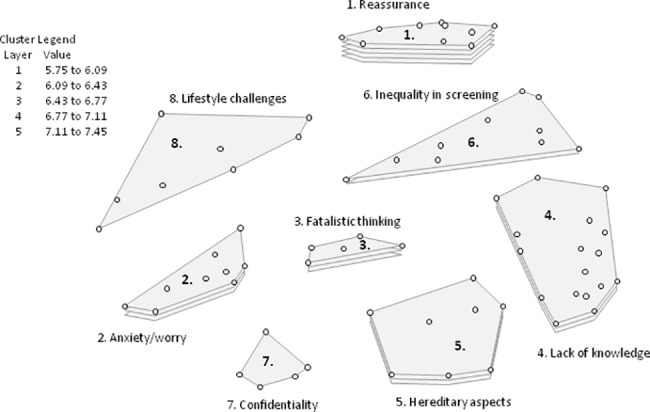
Cluster rating map Sweden^a^. ^a^Each cluster rating map displays clusters containing statements (the dots) that are similar in thematic content. The layer(s) of each cluster represents the average priority ratings of the statements within that cluster, more layers signify higher priority. The legend provides an indication of the average priority ratings that the statements within each cluster received.

Swedish participants generated between 3 and 15 categories (median = 11). Average statement ratings varied from 3.90 (statement: ‘why am I paying for high risk women to attend screening more frequently?’) to 9.00 (statement: ‘I want to be informed of both the benefits and the risks of personalised screening and prevention’). The overall fit of the concept map generated by the data of the Swedish participants was sufficient (stress-value = 0.29). Fig 3 presents the cluster rating map of the Swedish professionals.

The cluster rating map which provided the best fit contained eight categories: (1) Reassurance; (2) Anxiety/worry; (3) Fatalistic thinking; (4) Lack of knowledge; (5) Hereditary aspects; (6) Inequality in screening; (7) Confidentiality; and (8) Lifestyle challenges. The category with the highest average priority rating was ‘Reassurance’ (7.45), which includes statements such as: ‘I experience an increased sense of safety because I know my risk’ and ‘I feel reassured because additional methods will be used when mammography is not enough’. The categories ‘Confidentiality’ and ‘Lifestyle challenges’ were considered least relevant to women’s decision-making process with average rating scores of 5.75 and 5.76, respectively. ‘Confidentiality’ contains statements such as: ‘Will I lose my job if my employer knows I’m at high risk of developing breast cancer?’, ‘Will my employer get access to my risk estimate?’, and ‘Will my private health insurance premium increase?’. Examples of statements in ‘Guilt about lifestyle’ are: ‘I feel ashamed since I can’t get rid of my excess weight, which increases my risk of developing breast cancer’ and ‘I find it too difficult to change my lifestyle’.

A comparison of the rating patterns of Swedish clinicians (n = 6) versus other Swedish professionals (n = 6) showed moderate correlation (*r* = 0.60). Both groups prioritised ‘Reassurance’ in women’s decision-making. Clinicians further prioritised ‘Lack of knowledge’ and ‘ Hereditary aspects’, whereas other professionals prioritised ‘Fatalistic thinking’ and ‘Anxiety/worry’. Clinicians considered the theme of ‘Fatalistic thinking’ to be relatively unimportant in the decision-making process, rating it sixth out of seven, before ‘Lifestyle challenges’. Other professionals considered ‘Confidentiality’ the least important theme.

### Integration and cultural variation

Comparing the cluster rating maps of the three countries on thematic level, it transpires that the following five themes are prioritised by professionals: (1) Anxiety/worry; (2) Proactive approach; (3) Reassurance; (4) Lack of knowledge; and (5) Organisation of risk assessment and feedback.

Moreover, the themes show several differences in professionals’ perspectives. Both Dutch and British professionals considered voluntary participation relevant to women’s decision-making. Swedish professionals, however, did not prioritise this topic. Swedish professionals emphasised the potential impact of the new screening and prevention paradigm on family relations and the challenges involved in adopting lifestyle changes. Together with British professionals they also prioritised potential psychological consequences of risk feedback other than anxiety and worry, and emphasised women’s lack of knowledge. Furthermore, Dutch and Swedish professionals mentioned the heterogeneous nature of the new programme. Conclusively, only Dutch professionals identified communication, potential effects on screening outcomes and adherence, and stigma (i.e. personal responsibility for health) as themes which could influence decision-making.

## Discussion

The present study explored professionals’ perceptions of women’s decision-making regarding participation in personalised breast cancer screening and prevention. Distinct differences in perceptions were visible across professionals from the Netherlands, UK, and Sweden when evaluating the themes that were generated. However, these differences appeared less pronounced after evaluation of the generated statements on item-level, showing similar statements that were grouped and labelled differently depending on the country (S1 Table).

The central theme which was mentioned consistently by professionals of all three countries was ‘Anxiety/worry’. Professionals perceived the screening and prevention programme to induce anxiety about personal breast cancer risk, (late) detection of breast cancer, confrontation with an unhealthy lifestyle, chemoprevention, and the potential impact on relatives. This is in line with views expressed by women in previous research who described a concern for the potential impact of breast cancer risk information on their lives and their ability to manage subsequent decisions regarding screening and prevention [15]. The term ‘chemoprevention’ for risk-reducing medication in particular has been shown to elicit strong reactions through its perceived association with breast cancer and women’s reluctance to disrupt their current state of health, fearing debilitating side effects [16,21]. Additionally, although the assigned category labels may not automatically suggest this, professionals from all three countries generated statements about other potential psychological consequences which correspond to concerns mentioned by women in previous studies, e.g. false reassurance and fatalistic, obsessive thinking [14,16,21]. However, when categorising the statements and assigning category labels, professionals from each country used different words to describe these themes, which is reflected in the labels assigned by the software.

Besides ‘Anxiety/worry’ four other themes received high average priority ratings from professionals, i.e. ‘Proactive approach’, ‘Reassurance’, ‘Lack of knowledge’, and ‘Organisation of risk assessment and feedback’. The first three themes are in concordance with women’s perceptions of personalised breast cancer screening and prevention. Women have identified a proactive approach, perceived reassurance and knowledge as incentives to participate in the programme. They indicated that being proactive provides a sense of calm and perceived control [22–24]. Women related reassurance to the intensified screening and prevention programme that would be provided if they were above average risk [25]. Conversely, receiving a below average risk result provided no perceived reassurance [13,14]. Increased knowledge facilitated women’s acceptability of personalised screening and prevention, particularly of risk-reducing medication [8,17,25]. These similarities between professionals’ and women’s perceptions of factors that may influence participation will facilitate a shared decision-making process.

The priority assigned to the theme ‘Organisation of risk assessment and feedback’ appears to reflect professionals’ personal perspectives, since this topic was not previously mentioned by women. Statements in this category mainly relate to potential provider(s) of risk information and counselling, perceived accuracy of risk, and the pathway of care a woman will be assigned to based on her risk. These topics are also reflected in four other themes, i.e. ‘Defining and assessing risk’, ‘Effect on screening outcomes’, ‘Effect on screening adherence’, and ‘Concerns about accuracy’. Noticeably, these themes were mentioned by either Dutch or British professionals, but not by Swedish professionals. Participating professionals from NL and UK approached the acceptability of the programme with some scepticism, suggesting that women will question their personal risk information and their assigned pathway of care (e.g. screening frequency or biomedical prevention). Additionally, Dutch and British professionals fear more overdiagnosis and overtreatment, and expect an increase in opportunistic screening, particularly from women at below average risk. This perception is not supported by Evans and colleagues who relayed personal breast cancer risk information to women participating in the UK screening programme [26]. Women who were identified as below average risk were less likely to attend the following mammogram than women who were found to be at above average risk [26]. This may suggest that women at below average risk supported the idea of decreasing the screening frequency. Alternatively, it may also indicate that women experience a sense of false reassurance, considering the relatively wide screening interval in the UK of three years. It is important to address the concerns expressed by Dutch and British professionals before implementation, since previous research has shown that professionals who question the effectiveness of risk-based screening and prevention are less likely to breach the topic with eligible women [6,9,11]. Future research should further explore the acceptability of risk-based breast cancer screening and prevention from the perspective of eligible women, focusing on women’s views on breast cancer risk assessment, communication, and subsequent pathways of care (including potential opportunistic screening or lower screening intent).

Professionals appeared not to reference or prioritise risk communication; only Dutch professionals labelled a category accordingly. However, evaluation of the individual statements generated by participants of each country shows that all professionals explored some aspects of risk communication, e.g. provider or type of information. Insights in the topics that need to be discussed with eligible women is provided by a theme generated by both British and Swedish professionals labelled ‘Lack of knowledge’. This theme contains statements such as: ‘Does high risk mean I will develop breast cancer?’, ‘What alternative surveillance do I get if I am no longer offered screening?’, ‘How are my risk factors assessed?’, and ‘Are there other ways to reduce my risk of breast cancer?’. Professionals have previously indicated that risk information should be elaborate and presented in different formats [11]. Furthermore, they pointed out a need for additional training in risk communication to facilitate women’s understanding [27,28]. Suggested practice in risk communication encourages professionals to, e.g. avoid relative risks, encourage women to reflect on screening and preventive preferences from different perspectives, and to recognise that a woman’s decision may not reflect the professional’s preference [29].

### Strengths and limitations

A major strength of the present study is the participation of a wide variety of professionals from three European countries. This enabled cross-cultural comparisons in professionals’ perceptions of women’s decision-making regarding personalised breast cancer screening and prevention. However, due to the relatively small sample sizes per country it is difficult to conclusively ascertain whether the demonstrated differences are due to cultural variation or another reason, e.g. professional background. The British sample contained a relatively high proportion of clinicians compared with the Dutch and Swedish samples. This may have affected the integrated professional perspective, because clinicians generally have more contact with women than researchers, and may therefore have a clearer understanding of women’s perceptions.

The data analysis was performed by one researcher, however, methods were objectified by systematically assessing the thematic content of each cluster for the most general to the most specific content analysis as elaborated on in the methods section.

Concept mapping software enabled a time efficient, systematic approach to the analysis of qualitative data minimising individual interpretation bias, by analysing participants’ sorts and mean priority ratings. Consequently, the software relies heavily on the participant data, which will vary in quality. This can lead to suboptimal results, particularly in the cluster analysis, where participants’ categorisation of statements is averaged. It is conceivable that participants who generated relatively few categories negatively impact the analysis, by grouping statements together which are reasonably dissimilar in content. This has resulted in some categories containing statements that do not optimally match the assigned label (S1 Table). However, overall we believe the concept maps provide a relevant overview of themes that are potentially associated with women’s decision-making process regarding personalised breast cancer screening and prevention.

## Conclusion

The perspectives of Dutch, British, and Swedish professionals of women’s decision-making regarding personalised breast cancer screening and prevention generally appear in line with women’s own views of acceptability, which will facilitate shared decision-making. However, concerns regarding potential consequences of this new programme for screening outcomes and organisation need to be addressed, since this may affect how professionals communicate the programme to eligible women.

## Supporting information

S1 Table Overview of all generated clusters, statements and average priority ratings, stratified by country. (DOCX)
