# Supplementary material for: Women's decision-making regarding risk-stratified breast cancer screening and prevention from the perspective of international healthcare professionals
Source: PLoS One. 2018 Jun 1;13(6):e0197772. doi: 10.1371/journal.pone.0197772 (PMC5983562; doi:10.1371/journal.pone.0197772)
Supplement: S1 Table — (DOCX) [file pone.0197772.s001.docx]

**Appendix 1.** Overview of all generated clusters, statements and average priority ratings, stratified by country

| **The Netherlands** | | |
| --- | --- | --- |
| **Cluster name** | **Statement** | **Rating** |
| **1. Anxiety/worry** | | **7.23** |
|  | Knowing I’m high risk will cause a lot of worry and anxiety | 7.82 |
|  | If I know I’m high risk, I’ll be more afraid of getting breast cancer | 7.76 |
|  | Unrest can increase with personalised prevention, especially with women who are at high risk due to non modifiable factors | 7.76 |
|  | A longer screening interval induces anxiety | 7.59 |
|  | Knowing your breast cancer risk will induce anxiety | 7.59 |
|  | Women will be afraid to receive their risk result | 7.59 |
|  | Anxiety will increase if the high risk is mostly due to genetic factors | 7.53 |
|  | Risk information will increase anxiety | 7.41 |
|  | Receiving a high result will induce anxiety | 7.35 |
|  | If I receive less screening, my breast cancer will not be detected on time | 7.24 |
|  | I don’t want to know my breast cancer risk | 6.82 |
|  | More mammograms will give you increased anxiety every time | 6.47 |
|  | I worry about a confrontation with my unhealthy lifestyle | 6.00 |
| **2. Attitude to risk** | | **6.33** |
|  | I appreciate the opportunity to detect a breast cancer earlier | 8.53 |
|  | It’s nice to know that you have a low risk of developing breast cancer | 7.41 |
|  | Once I know I have a low risk of developing breast cancer, I’ll be reassured | 7.35 |
|  | I like knowing that I am in the low risk category | 7.29 |
|  | I’d like to know my risk | 7.24 |
|  | Personalised screening makes women responsible for difficult choices regarding risk feedback and prevention | 7.24 |
|  | Knowing your risk is reassuring | 6.82 |
|  | Knowing your risk makes you worry about insurance and the government | 6.76 |
|  | I don’t want to know that I have a high risk of developing breast cancer | 6.59 |
|  | Knowing your risk provides stress, because there are too many options | 6.53 |
|  | I don’t want to know my risk, because that would worry me too much | 5.76 |
|  | Having a mammogram done every two years is bad enough | 5.47 |
|  | Having high risk gives the impression that you are ill | 5.41 |
|  | Providing a blood sample is more stressful than a mammogram | 3.41 |
|  | If I don’t think about it, it’s not actually happening | 3.18 |
| **3. Defining and assessing risk** | | **6.04** |
|  | If my risk is higher than that of the average Dutch woman, then I want better early detection and preventative options. | 8.12 |
|  | It is important to determine what we will base our criteria of risk on | 7.47 |
|  | Personalised screening should be an addition, not a replacement; low risk women should still receive frequent screening | 7.41 |
|  | Personalised screening medicalises a woman’s life | 7.18 |
|  | If the woman has an increased risk of developing breast cancer, she would rather receive surveillance in a hospital | 7.18 |
|  | Communicating risk information is difficult; it’s vague and leading | 7.06 |
|  | A personal risk profile should determine the screening age range | 6.82 |
|  | Women who border the cut-off for high risk want to receive the strategy that high risk women get | 6.59 |
|  | If I’m high risk, does that mean that my daughter is as well? | 6.53 |
|  | Not everyone understands the meaning of risk which complicates informed decision-making | 6.53 |
|  | A risk estimate is still an estimate; you never know whether you’ll get breast cancer or not | 6.24 |
|  | Personalised screening disables low risk women to detect a breast cancer early | 6.00 |
|  | Screening is mostly useful if you have a family history of breast cancer | 5.82 |
|  | Women want alternatives to mammograms | 5.71 |
|  | Every risk is a risk, no matter how low | 5.18 |
|  | The woman disagrees with the risk criteria and her risk result | 4.88 |
|  | It’s better to be high risk, because you get extra screening | 4.88 |
|  | This programme is good, because it is initiated by the government | 4.59 |
|  | All women should be tested before they are thirty | 4.59 |
|  | Personalised screening will reduce the number of unnecessary biopsies | 4.06 |
|  | A blood test feels more medical than a mammogram | 3.94 |
| **4. Effect on screening outcomes** | | **5.88** |
|  | A high risk woman will want additional tests | 7.35 |
|  | It is difficult to understand a risk result | 7.06 |
|  | Screening is an important check-up, I want it as frequently as possible to feel reassured | 7.06 |
|  | I don’t want to receive less screening, because what if they got the risk estimate wrong | 7.00 |
|  | By screening high risk women more frequently, you increase the chances of false positive outcomes and overdiagnosis | 6.29 |
|  | I would prefer to be screened each year, because this facilitates early detection | 6.00 |
|  | This will increase the number of worrying mammograms | 5.88 |
|  | Does early detection still work if you screen less frequently? | 5.76 |
|  | High risk women will demand additional unnecessary care | 5.53 |
|  | More screening will increase my exposure to radiation, which will further increase my breast cancer risk | 5.12 |
|  | Women will be insecure about providing the correct answers to the questionnaire on which their risk will be based | 4.94 |
|  | Personalised screening will increase the number of unnecessary biopsies | 4.59 |
|  | Personalised screening will reduce the number of worrying mammograms | 3.88 |
| **5. Communicating risk** | | **5.22** |
|  | A personalised risk estimate will lead to a personalised prevention/lifestyle advice | 8.06 |
|  | Excellent that we are trying everything within our power to reduce risk | 7.06 |
|  | Information on preventative measures is very ambiguous | 6.18 |
|  | Personalised screening can only be effective if high risk women adhere to the prescribed preventative measures | 6.00 |
|  | You can’t keep adjusting your lifestyle to prevent disease | 5.35 |
|  | Information about preventative measures in the general population should be relayed by general practitioners | 5.00 |
|  | Prevention with medication can lead to side-effects | 4.65 |
|  | Women don’t always report their risk information correctly | 4.35 |
|  | Information about preventative measures in the general population should be relayed by clinicians | 4.12 |
|  | I don’t want to change my lifestyle, but if I can reduce my risk with a pill then that’s great | 3.53 |
|  | This programme is mostly useful for women with a bad lifestyle | 3.12 |
| **6. Proactive approach & stigma** | | **6.64** |
|  | Knowing you’re high risk enables you to act accordingly | 7.82 |
|  | It’s right that women at high risk receive information on how to lower their risk | 7.41 |
|  | I like knowing that I’m high risk, because I can take measures into my own hands trying to reduce my risk | 7.35 |
|  | Education on primary prevention offers women the opportunity to decrease their risk | 7.24 |
|  | It’s right that I’ll be given advice on how to improve my lifestyle | 6.94 |
|  | By actively changing some of my risk factors, I can reduce my personal risk of developing breast cancer | 6.82 |
|  | It is difficult to change your lifestyle | 6.29 |
|  | I will gladly eat less and exercise more, but I will not medicate a healthy body | 5.76 |
|  | Personalised screening makes women personally responsible for the development of breast cancer | 5.47 |
|  | Women will have to travel further to a screening unit due to higher demand | 5.29 |
| **7. Screening inequality** | | **5.99** |
|  | A high risk woman will want a decreased screening interval | 8.35 |
|  | It is right that high risk women will be invited for screening more often | 7.76 |
|  | I like that my personal circumstances will be taken into account | 7.41 |
|  | It is difficult to understand risk information | 6.65 |
|  | It is difficult to understand that my neighbour received a different screening strategy to me | 6.59 |
|  | Personalised screening makes me feel more like an individual, rather than part of a large group of women | 6.59 |
|  | I like that I receive personal attention | 6.24 |
|  | Personalised screening makes me feel more in control of the value of screening for me personally | 6.24 |
|  | Personalised screening provides reassurance | 6.00 |
|  | Personalised screening will provide false reassurance to women at low risk | 5.71 |
|  | Personalised screening provides false reassurance regardless of risk | 5.65 |
|  | It is my right to receive screening | 5.41 |
|  | If the government says I don’t need screening as frequently, then they are probably right | 5.24 |
|  | A low risk woman will want an increased screening interval | 5.18 |
|  | I know I’m not high risk, no matter what the test result says | 4.35 |
|  | Personalised screening results in an unequal division of preventative services | 4.29 |
|  | It is unfair that high risk women are invited more frequently than low risk women | 4.12 |
| **8. Freedom of choice** | | **5.48** |
|  | Personalised screening will motivate high risk women to attend screening | 8.18 |
|  | Knowing your risk enables you to make an informed decision about your participation in screening programmes | 7.00 |
|  | It is unfair that only high risk women are informed about ways to reduce their risk | 6.47 |
|  | Women who participate in screening already struggle to understand the information leaflet; more complex information will be too much | 6.41 |
|  | I used to be screened biennially, but now suddenly I’m not, because I’m low risk | 6.35 |
|  | You feel forced to behave proactively | 5.88 |
|  | I will not take the medication. First I need to know for sure that it’s safe and doesn’t cause other diseases | 5.12 |
|  | I don’t need to participate in primary prevention; I feel well | 4.88 |
|  | I don’t need more frequent screening; I feel well | 4.76 |
|  | My personal identity will be dominated by risks | 4.76 |
|  | I will not participate in primary prevention; you’re not allowed to do anything anymore these days | 4.65 |
|  | I live a healthy life; that should be sufficient | 4.53 |
|  | Great that I can influence my risk factors, this means I no longer need to attend screening | 4.53 |
|  | I object to storing DNA information | 4.47 |
|  | I object to sharing information about my health | 4.18 |
| **9. Effect on screening adherence** | | **5.50** |
|  | Women who find screening painful, but now know they’re high risk, will increase their attendance | 6.71 |
|  | More frequent screening is a burden | 6.71 |
|  | It is comforting to know where I stand with regards to risk | 6.47 |
|  | A mammogram is painful and I will therefore attend as infrequently as possible | 5.88 |
|  | Low risk women will avoid screening | 5.41 |
|  | If the woman does not perceive any breast cancer risk, she will no longer regard screening as useful | 5.29 |
|  | If I know I’m low risk, I don’t need to attend screening | 5.24 |
|  | If my risk is lower than that of the average Dutch female, then I don’t have to attend screening | 4.76 |
|  | Who has the time to participate in more frequent screening?! | 3.06 |
| **The United Kingdom** | | |
| **Cluster** | **Statement** | **Rating** |
| **1. Organisation of risk assessment and feedback** | | **7.05** |
|  | Does low risk mean I won't develop breast cancer? | 8.40 |
|  | Will I get other types of screening if I am at higher risk? | 8.33 |
|  | I would like to discuss my risk and options with a doctor or nurse. | 8.14 |
|  | I would like to discuss my risk with someone. | 8.13 |
|  | I do not understand the information given in my initial risk letter. | 7.53 |
|  | My sister died of breast cancer but my risk is supposed to only be average. | 7.47 |
|  | Who will decide whether I get more or less frequent screening? | 7.47 |
|  | Can I get more frequent screening if I want it? | 7.36 |
|  | Who will advise me about options to reduce my risk? | 7.29 |
|  | Why can't I have extra screening just because I'm average risk? | 7.00 |
|  | Does low risk mean I don't need to go for screening? | 6.80 |
|  | How will risk and primary prevention options be communicated to me? | 6.80 |
|  | Will I get NHS breast screening or do I have to pay for private screening? | 6.80 |
|  | What if I disagree with the risk assigned to me? | 6.73 |
|  | Who knows about my risk? | 6.67 |
|  | When are my risk factors assessed? | 6.43 |
|  | Why don't they screen younger and older women? | 5.57 |
|  | Is this done in other countries? | 4.00 |
| **2. Lack of knowledge** | | **7.20** |
|  | Does high risk mean I will develop breast cancer? | 8.73 |
|  | Are there other ways to reduce my risk of breast cancer? | 8.07 |
|  | Why is my risk higher than my sister's? | 8.00 |
|  | What options are available to me to reduce my risk? | 7.87 |
|  | I am high risk, do my sisters need to be tested? | 7.80 |
|  | What are the risk factors that mean I get more frequent screening? | 7.73 |
|  | I do not know enough about benefits/risks of this new prevention programme. | 7.73 |
|  | Can I change my risk? | 7.53 |
|  | What does risk estimation involve? | 7.47 |
|  | How will they know if my risk changes? | 7.33 |
|  | It would help if you could make the whole process of risk estimation simple. | 7.27 |
|  | How are my risk factors assessed? | 7.13 |
|  | How do I know that exercise or diet will help reduce my risk of breast cancer? | 7.13 |
|  | Why is my risk higher than my friend's? | 7.00 |
|  | My friend only has to have mammograms every 3 years; why do I have to have them more often? | 6.93 |
|  | What if I struggle to lose weight; will I definitely get breast cancer? | 6.64 |
|  | My friend is getting mammograms every 18 months, why can't I? | 6.53 |
|  | At what age would my risk be assessed? | 5.93 |
|  | Doctors go on about weight, but I have read that being overweight is OK and in some women decreases risk of breast cancer. | 5.80 |
|  | I want online access to programmes. | 5.47 |
| **3. Concerns about accuracy** | | **6.88** |
|  | I know a number of women who have breast cancer without any risk factors, we are all at risk. | 7.60 |
|  | I have read that a lot of cancers are detected which would never cause me any problem in my lifetime. | 7.47 |
|  | I'm at low risk so I don't really need to come for any more mammograms. | 7.13 |
|  | My risk may not be accurate, e.g. if I'm unaware of my family history there's no point in finding out as the information won't be accurate. | 6.33 |
|  | I am high risk and I feel I should proceed to surgery. | 5.87 |
| **4. Anxiety/worry** | | **7.40** |
|  | I am high risk and I am now worried about my daughters. | 8.87 |
|  | I don't want to stop screening. | 8.53 |
|  | I want the chance to discuss what I might do about my risk. | 8.53 |
|  | If I am screened less often I will worry that something is more likely to be missed. | 8.47 |
|  | Being removed from the screening programme might be scary. | 8.27 |
|  | I don't want to know if my risk is high as this will cause too much worry. | 8.00 |
|  | I do not want to reduce my screening frequency; this worries me. | 7.73 |
|  | Risk-reducing medication has a lot of side effects; this makes me worried. | 7.60 |
|  | I wonder whether more frequent screening/ radiation exposure will add to my risk. | 7.40 |
|  | I'm concerned about communicating risk and prevention choices with other family members. | 7.33 |
|  | Perhaps more likely to want to know if they think they will be low risk. | 6.67 |
|  | The doctors do not want me to proceed to surgery, instead they are offering me tablets; this worries me. | 6.33 |
|  | I want my risk re-assessed because I don't believe the result. | 6.27 |
|  | What if I struggle to do more exercise; will I definitely get breast cancer? | 6.20 |
|  | Tamoxifen is a cancer drug. | 4.87 |
| **5. The psychological impact of knowing risk** | | **7.06** |
|  | Knowing my individual risk makes me anxious. | 8.53 |
|  | Women might become worried and anxious if they assumed that they were at an average risk, but then are told they are high risk. | 8.53 |
|  | I feel anxious with personalised screening as I am more aware of my risk. | 8.27 |
|  | If I am screened more often I will be more anxious waiting for results from the screening. | 8.27 |
|  | Women may become paranoid rather than reassured with medical input. | 8.07 |
|  | Personalised screening has made me obsess about my breast cancer risk. | 7.73 |
|  | I have increased screening so I feel like I'm just waiting to get cancer. | 6.93 |
|  | I am afraid of being labelled as poor health by those around me. | 6.67 |
|  | I fear that someday knowing risk could affect my life insurance. | 6.60 |
|  | I worry that people will criticise my lifestyle, e.g. drinking too much. | 6.57 |
|  | I will feel and behave as if I'm unwell, even though I am not. | 6.53 |
|  | I am afraid of doctors. | 6.29 |
|  | I am worried about the costs of additional mammograms and preventive options. | 5.93 |
|  | I feel guilty that I'm unable to keep up with the lifestyle change. | 5.93 |
|  | My friends might know if I start taking a pill. | 4.93 |
| **6. Proactive approach** | | **7.76** |
|  | This may help my children or relatives; if my risk is high, theirs could be too. | 9.13 |
|  | I am very keen to have additional mammograms if I am at high risk. | 8.93 |
|  | I want to do everything you can to avoid breast cancer. | 8.73 |
|  | This new programme gives me peace of mind. | 8.69 |
|  | It's a comfort to know that I'm being thoroughly checked out. | 8.60 |
|  | I can be more aware of potential symptoms if my risk is high. | 8.53 |
|  | I feel positive about identifying cancer sooner with personalised screening. | 8.33 |
|  | I feel empowered being part of a personalised screening programme. | 8.33 |
|  | I can take control of some of my own risk factors and take steps to reduce them. | 8.33 |
|  | This sounds very sensible; I don't want to have extra mammograms if they are not necessary. | 8.33 |
|  | I feel more looked after with personalised screening that is holistic to my needs. | 8.20 |
|  | Personalised screening is better for me and will reduce my risk. | 8.13 |
|  | I am pleased that I am low risk and that I do not need frequent mammography. | 8.13 |
|  | I would greatly appreciate a supervised diet/weight programme to reduce risk. | 7.87 |
|  | I want to see a positive outcome of going along with the plan. | 7.73 |
|  | I want easy access to tests (times and places). | 7.14 |
|  | I am keen to explore ways of reducing weight. | 6.93 |
|  | I am low risk so I will attend screening more consistently, because otherwise it would be too long till my next mammogram. | 6.27 |
|  | This screening programme is not accessible to all women, for example lower income women may not attend. | 4.73 |
|  | I want to be able to interact with other participants. | 4.20 |
| **7. Acceptance of risk without intervention** | | **6.53** |
|  | I do not want to know my risk of developing breast cancer. | 7.33 |
|  | I don't feel any extra benefit from knowing risk; I won't change my behaviour so what is the point. | 7.33 |
|  | I don't want more screening if I'm high risk as it is unpleasant/painful. | 7.27 |
|  | I can't change it anyway, so why find out what the risk is? | 7.27 |
|  | I do not want to try and reduce my risk of breast cancer. | 7.15 |
|  | I think that mammograms cause breast cancer anyway so I do not want to have more. | 7.13 |
|  | I just want to have a mammogram and not be worried about all these other things. | 7.00 |
|  | I just want to live a normal life. | 6.87 |
|  | I don't have time to take part in a personalised risk screening programme. | 6.43 |
|  | I feel its fate and I don't want to know my risk. | 6.40 |
|  | I can't be bothered with keeping up e.g. exercise programme. | 6.13 |
|  | I do not like taking pills. | 6.00 |
|  | I don't have enough time to change my risk. | 6.00 |
|  | I don't want to give blood or a saliva sample to determine my risk. | 5.87 |
|  | I do not want to reduce my alcohol intake. | 5.73 |
|  | I will not develop breast cancer, no matter what my risk is. | 4.67 |
| **Sweden** | | |
| **Cluster** | **Statement** | **Rating** |
| **1. Confidentiality** | | **5.75** |
|  | I worry about the side effects of risk-reducing medication. | 7.92 |
|  | How should I tell my partner/family about my breast cancer risk? | 6.00 |
|  | Will I lose my job if my employer knows I have a high risk of getting cancer? | 5.25 |
|  | Will my private health insurance premium go up? | 4.90 |
|  | Will my employer get access to my risk estimates? | 4.58 |
| **2. Anxiety/worry** | | **6.56** |
|  | Knowing my risk makes me worried. | 8.08 |
|  | I worry about the consequences of taking drugs like tamoxifen. | 7.75 |
|  | I am high risk and fear cancer and dying young. | 7.33 |
|  | I will have more anxiety, because I will receive a mammogram result more often (high risk). | 6.70 |
|  | I get scared if my risk is higher than my friends' and I need to go to screening more often. | 6.67 |
|  | I want someone to take care of me if I get a "bad" result and need support. | 6.55 |
|  | I worry that insurance companies, society, or my workplace will stigmatise me if I'm high risk. | 5.83 |
|  | I will worry about cancer, even though I am not ill. | 5.75 |
|  | I will feel guilty towards myself, my partner/family if I fail to change my lifestyle. | 4.42 |
| **3. Fatalistic thinking** | | **6.52** |
|  | I perceive my breasts as a ticking bomb, because I am high risk. | 7.60 |
|  | It is difficult to accept that I need screening more often than others; why me? | 6.80 |
|  | I am very worried about my dense breasts; I am thinking of having them surgically removed! | 6.50 |
|  | I worry more about developing cancer, because of more frequent screening (for higher-risk women). | 6.00 |
|  | I worry more about developing cancer, because of less frequent screening (for low-risk women). | 5.92 |
| **4. Lifestyle challenges** | | **5.76** |
|  | I do not want to know my risk. | 7.92 |
|  | I do not want to pay additional fees for personalised screening. | 6.55 |
|  | I do not want to fill-out complex questionnaires for personalised screening. | 6.08 |
|  | Prevention is only going to take up time in my already hectic life. | 6.00 |
|  | I worry that my own behaviour/life style might be unhealthy. | 5.70 |
|  | I do not want to participate, because if I get breast cancer, it is the will of god. | 5.42 |
|  | I feel ashamed since I cannot stop smoking and I have been told that that gives me a higher risk of breast cancer. | 5.33 |
|  | I feel ashamed since I cannot stop drinking too much and I now know that increases my breast cancer risk. | 4.90 |
|  | I feel ashamed since I cannot get rid of my excess weight that gives me a higher risk of breast cancer. | 4.83 |
|  | I find it too difficult to change my lifestyle. | 4.60 |
| **5. Reassurance** | | **7.45** |
|  | I experience improved possibilities to detect cancer at an early stage. | 8.33 |
|  | Personalised screening is safer, because my risk factors are taken into account. | 8.10 |
|  | I am happy that I receive more check-ups. | 8.00 |
|  | I am happy to skip unnecessary mammography (for the low risk). | 8.00 |
|  | I would like to know my risk. | 7.83 |
|  | I experience increased safety, because I know my risk. | 7.83 |
|  | Better compliance to screening because of the individual approach. | 7.17 |
|  | I feel reassured because additional methods will be used when mammography is not enough. | 7.09 |
|  | I am low risk, therefore I do not need screening or other forms of prevention. | 6.00 |
|  | I feel content because I feel seen by the national healthcare programme. | 5.80 |
| **6. Inequality in screening** | | **6.18** |
|  | I will have a better prognosis with a high risk, because my cancer will be found earlier. | 7.83 |
|  | Problems with my health will be discovered earlier if I go to screening. | 7.83 |
|  | I want to be seen as an individual and not just as someone in a group. | 7.33 |
|  | My low risk still worries me and I do not accept less frequent screening. | 6.92 |
|  | If I am considered low risk, I won't have to worry about getting breast cancer, ever. | 6.91 |
|  | Everyone should have equal access to personalised screening. | 6.17 |
|  | I do not want to travel a long way to get my extra mammogram. | 5.58 |
|  | I will not accept barriers to participation, e.g. waiting lists. | 4.83 |
|  | Angelina Jolie also had a prophylactic mastectomy, so it is a good choice. | 4.58 |
|  | I do not like that my friend receives more screening than me. | 3.92 |
| **7. Lack of knowledge** | | **6.76** |
|  | I want to be informed about both the benefits and the risks of personalised screening. | 9.00 |
|  | I do not think that a 5-year screening interval is safe. | 7.83 |
|  | I am very confused about my risk and the consequences. | 7.80 |
|  | I am only screened every 5 years; what happens if I develop cancer in the meantime despite my low risk? | 7.50 |
|  | Is ultrasound (or MRI, or tomosynthesis, etc.) as good as a mammogram? | 7.17 |
|  | How accurate are the genetic tests? | 6.90 |
|  | How dangerous is it to be screened every year? | 6.83 |
|  | What primary prevention options do I have? | 6.75 |
|  | I do not believe my risk is correct. | 6.75 |
|  | What alternative surveillance do I get if I am no longer offered screening? | 6.64 |
|  | I would like more knowledge on healthy lifestyles. | 6.42 |
|  | I do not understand this new screening programme. | 6.25 |
|  | I do not believe that lifestyle changes will really lower my breast cancer risk. | 6.20 |
|  | Do I get all the test results? | 6.10 |
|  | I do not want more screening; if I get breast cancer I will notice and seek care. | 5.82 |
|  | What exactly are they testing with my blood? | 5.55 |
|  | Make sure that the screening personnel knows this examination is not a routine for the women, even if it is for them. | 4.90 |
| **8. Hereditary aspects** | | **6.52** |
|  | Is my daughter's risk also increased or decreased? | 8.67 |
|  | I am high risk, but I do not understand the consequences and therefore feel insecure. | 8.30 |
|  | Should my family members be screened too if I'm high risk? | 8.20 |
|  | I do not trust the reliability of the risk-assessment; this makes me worry. | 6.70 |
|  | I have given a lot of detailed information about myself; where does all this information end up and where is it stored? | 6.25 |
|  | Should I speak to my partner/family if I am at high risk of getting cancer? | 6.08 |
|  | I worry about receiving more screening because of the higher radiation dose. | 5.50 |
|  | Do I still want to have children if I belong to the high risk group? | 4.80 |
|  | Why am I paying for high risk women to attend screening more frequently? | 3.90 |
